# Categorical and Dimensional Diagnoses of Dyslexia: Are They Compatible?

**DOI:** 10.3389/fpsyg.2020.02171

**Published:** 2020-08-28

**Authors:** Luca Cilibrasi, Ianthi Tsimpli

**Affiliations:** ^1^Faculty of Arts, Charles University, Prague, Czechia; ^2^Department of Theoretical and Applied Linguistics, University of Cambridge, Cambridge, United Kingdom

**Keywords:** dyslexia, poor reading, categorical, dimensional, phonology, lexicon

## Abstract

Dyslexia is often assessed using categorical diagnoses, and subtypes of dyslexia are also recognized in a categorical fashion. Children may meet the criteria for dyslexia, and they may more specifically meet the criteria for a subtype of it, and thus get a diagnosis. This approach to diagnosis clashes with the actual distribution of reading performance in children (which is normal and continuous), and it has received criticism. This article offers a conceptual framework for conciliating these two positions. In short, the proposal is to use a set of multicomponent continuous assessments of reading, rather than thresholds. The proposal is explained using original data obtained from a sample of 30 children (age 7 to 11), tested in the United Kingdom. Using an assessment based on categorical-thresholds, only five children in our sample qualify for extra assistance, and only one may get a diagnosis of dyslexia, while with the mixed system proposed, a few additional children in the gray area would receive attention. This approach would not discard previous categorical approaches such as those distinguishing between surface and phonological dyslexia, but it would rather see these subtypes of dyslexia as the instance of a lower score on the continuum obtained on a single component of the multicomponent assessment.

## Introduction

The National Institute of Neurological Disorders defines dyslexia as “a brain-based type of learning disability that specifically impairs a person’s ability to read. Individuals (with dyslexia) typically read at levels significantly lower than expected despite having normal intelligence. Although the disorder varies from person to person, common characteristics among people with dyslexia are difficulties with phonological processing (the manipulation of sounds), spelling, and/or rapid visual-verbal responding”.

The brains of children with dyslexia show peculiarities that can affect various regions, including for example the visual word form area (Dehaene and Cohen, 2011), Broca’s area (Paulesu et al., 2001), the magnocellular pathway (Stein, 2001) and the cerebellum (Nicolson et al., 2001). However, these patterns are not consistent across subjects (Kraft et al., 2016; Łuniewska et al., 2019), and thus behavioral tasks remain the most effective assessment available (Fletcher, 2009; Ramus et al., 2018).

The transformation of text into sound is a complex process that involves various sub-skills, arguably phonological skills (Van Orden and Kloos, 2005), visual skills (Kulp and Schmidt, 1996), working memory (Siegel, 1994), and the coordination between them (Church et al., 2008). According to one of the most influential models of reading, the Dual Route Model of Reading (Coltheart et al., 2001), reading takes place through two systems simultaneously. On the one side, the Grapheme-Phoneme Conversion System, or non-lexical route, transforms individual graphemes into phonemes; on the other side, in the lexical route, strings of graphemes that would be unpredictable with the non-lexical route are associated with the pronunciation of entire words, recurring to stored information from the lexicon. Children can show dissociated disorders for certain scripts (Friedmann and Coltheart, 2018), since they might have difficulties either with transforming graphemes into phonemes (and thus struggle more with transparent orthographies, at least in the initial stages of learning, Rapcsak et al., 2009; Verhoeven and Keuning, 2018), or transforming entire words into sounds (and thus struggle more with opaque orthographies, Patterson et al., 2017). This variation can be exemplified comparing languages such as Czech and traditional Mandarin Chinese, which represent two extremes. While in Czech the pronunciation of words can be predicted very reliably using the graphic symbols (letters and additional signs around them), in Mandarin this is not possible, since symbols do not map into actual phonemes, but rather into entire words. The present study focuses on English, a language that contains elements from both systems (i.e., while some words can be read using grapheme-phoneme conversion, others must be read using the lexical route).

Over the years, researchers have been developing more and more refined behavioral assessments for dyslexia, focusing on various subcomponents of reading, and adjusting them depending on the orthography of the language used. Two sub-skills that seem to be reliable measures to detect a reading disorder are reading fluency (Meisinger et al., 2010) and decoding (Snowling et al., 2009). The first measure refers to the speed at which text is read, while the second one refers to the accuracy with which words are transformed into speech. In English, difficulties in decoding (particularly with non-words) are associated with a deficit in the phonological route, while difficulties with fluency are associated with a deficit in the lexical route (Wolf and Bowers, 2000). Current diagnoses of dyslexia rely on categorical assessments of these routes. If a child performs below a certain threshold in one or more of these subcomponents, they may receive a diagnosis.

## Methods

### Research Questions

Does the categorical approach fully capture children’s performance, and is it effective in identifying children with difficulties? Are there children who do not qualify for special assistance using a categorical approach, but that do so using a continuous approach? Can the two systems be conciliated?

### Design

A group of 30 children in two schools in the Cambridge (United Kingdom) area were assessed with a standardized reading task. The classification based on the standardized task was then compared to an interpretation of the same data based on the multicomponent “mixed” approach.

### Ethics, Recruitment, and Consent

The current study received ethical approval from the University of Cambridge Ethics Committee. Several schools in the area were contacted and invited to participate. Two schools decided to participate, and parents were provided with information sheets and consent forms by the schoolteachers. Thirty children finally participated in the study. Children were aged between 7 and 11, 16 were male and 14 were female, and none had a diagnosis of language or developmental impairments.

### Materials

Children were assessed with a battery of standardized tasks. These are: The York Assessment of Reading and Comprehension – YARC (Snowling et al., 2009), the Colored Progressive Matrices – CPM (Raven, 1962), and the Children’s Test of Non-word Repetition – CNRep (Gathercole et al., 1994).

The CNRep is a widely used non-word repetition task, aimed at assessing verbal working memory. The test is composed of 40 non-words of different length (10 two-syllable non-words, 10 three-syllable non-words, 10 four-syllable non-words, and 10 five-syllable non-words). All children performed within norms for their age in this task.

The CPM is a widely used non-verbal intelligence task in which, in each trial, children are required to complete a puzzle choosing between six different figures. The correct figure matches the geometric pattern of the main figure, while the other five do not. Children were presented with sections A and B (24 trials). All children performed within norms for their age in this task.

The YARC is the main task in this study. It is a reading assessment in which children are asked to read short texts and are then assessed with comprehension questions. While the child reads, the researcher identifies decoding mistakes and measures the time the child needs to read. The task provides thus three outcome measures: decoding, speed and comprehension.

The socioeconomic status of children in this sample was either at or above the national average. In terms of catchment area, one school is in a lower-middle-class district of the Cambridgeshire County, one in an upper-middle-class district^1^.

### Procedure

Children were assessed one by one by the researcher in a quiet room. Most of the children were tested during school time in the school premises. Some parents preferred to bring the children to the department in the afternoon, and children were thus tested in the Psycholinguistics Lab of the Department of Theoretical and Applied Linguistics, University of Cambridge.

## Results

The report of the descriptive statistics for age and all of the tasks (Table 1).

**TABLE 1:** Descriptive statistics.

	**Age (in months)**	**CNRep (# of errors)**	**CPM (# of errors)**	**YARC D. (percentile)**	**YARC R. (percentile)**	**YARC C. (percentile)**
**Mean**	111	7.2	3.9	55.3	53.2	68.2
**SD**	14	3.5	2.5	29.9	31.3	23.2

*YARC D, YARC decoding; YARC R, YARC rate, YARC C, YARC comprehension.*

The next section will present two analyses of the sample, one categorical and one “mixed” (containing elements from both a categorical and a dimensional approach). No statistics are performed on the data since each child is treated as a potential client. In other words, the analysis does not assess the sample as an entity but rather treats each individual in the sample as a subject that may or may not get a diagnosis, if assessed. In a clinical setting, the assessment of an individual child would not include any statistical analysis, and this is thus the choice adopted for the subjects in this sample.

### A – Categorical Assessment

Table 2 presents the individual percentile scores on the York Assessment of Reading and Comprehension. The YARC manual provides guidelines on the classification of the percentiles. The guidelines of the YARC use a division that is intrinsically categorical, though not binary. The YARC offers, in fact, five levels of performance, and two levels of impairment: *severe difficulty* and *below average* (Snowling et al., 2009, p. 57):

**TABLE 2:** Individual percentiles in the three sections of the task.

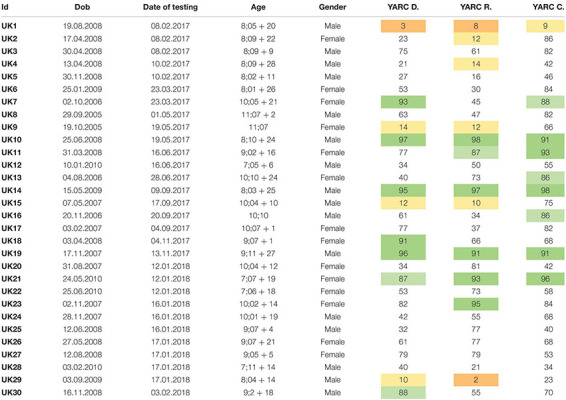

*Dark orange, severe difficulty; light orange, below average; white, average; light green, above average; dark green, excellent; following the YARC Manual (Snowling et al., 2009).*

1. Children with a percentile of 8 or below are identified as having severe difficulty
2. Children with a percentile between 9 and 14 are identified as below average
3. Children with a percentile between 16 and 84 are identified as average
4. Children with a percentile 86 and 91 are identified as above average
5. Children with a percentile above 92 are identified as excellent

Even if the YARC’s authors are rather cautious about the use of this classification, the manual does offer some guidelines on the practical implications of the scores, developed following the government policies of the United Kingdom. Providing some examples, chapter 6 of the manual reports on the use of the task with special populations: Case 2 (Snowling et al., 2009, p. 78) is that of a child with dyslexia. As the authors explain, below-average performance in decoding and rate is associated with dyslexia.

“J. displays some weaknesses with decoding skills; reading accuracy and reading rate are below average for her age. Her reading comprehension skills are average for her age. This profile is often associated with dyslexia.”

In the sample presented in this study, this same pattern would apply to UK1. Page 8 of the manual (Snowling et al., 2009) is more general (it does not use specific examples), and it states that following the government guidelines children should be granted extra time in written work if they are *below average* in decoding and/or rate.

“At the time of writing, it is reasonable to expect that pupils may be eligible for additional time if they fulfill one or both of the following criteria: (1) Reading accuracy score is below average for their age; (2) Reading rate score is below average for their age.”

In the sample presented in this study, this would apply to UK1, UK2, UK4, UK9, UK15, and UK29. As we will explain in the next section, this approach excludes some children that may benefit from extra time, or from additional assistance.

### B – Mixed Assessment

**FIGURE 1:**
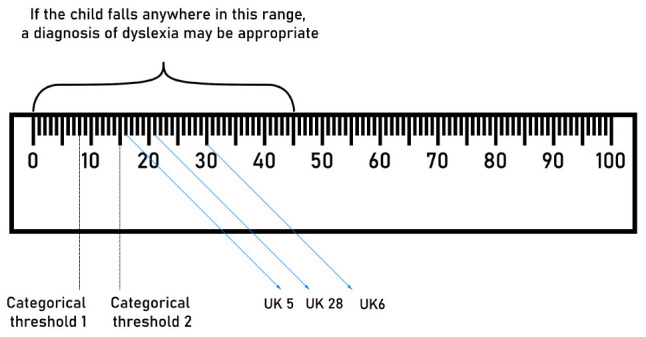
Chart combining categorical and dimensional approach. Some children in the “gray area” from our sample are reported.

In Figure 1, we report a chart that provides a guideline on how to obtain a mixed assessment. Clinicians may follow this simple procedure to get an assessment that conciliates a categorical and a dimensional approach:

If a child falls below threshold 2, a diagnosis may be granted without additional questions.

If a child falls above the threshold, but anywhere in the bottom half of the distribution, a diagnosis shall still be possible, following the evaluation of additional parameters.

The assessment of children in the gray area is the crucial advantage of a mixed approach. As a general guideline, a diagnosis should become less likely the higher the score in the gray area, but what procedures should the clinicians follow to make a decision for children with an unclear outcome? As Protopapas (2019) notices, a paradigmatic change on the treatment of dyslexia as a continuum must rely on the sensitivity of the clinicians performing the assessment. Clinicians should take into account various parameters for all of the children in gray areas of the distribution, the most important one being whether the child appears to need additional assistance, or not. This idea is captured in this quote from Protopapas (2019):

“If you admit that dyslexia is continuous with the general population then gray areas and ambiguities are inherently expected. […] This requires the exercise of more judgments on behalf of the clinician; but this is why we need expert, well-trained clinicians, so they can exercise their judgment wisely and toward the benefit of the children under diverse circumstances and resource pressures.”

In our sample, a mixed approach brings attention to a few cases that would not be considered with a categorical approach. UK5 has a reading rate in the 16th percentile (quite low, but not below the threshold). UK28, similarly, has a reading rate in the 21st percentile (again, quite low, but not below the threshold). If one compares UK2 and UK28 (for example), it is possible to see the limits of the categorical approach. UK2 is a slow reader, but has a high performance in comprehension, and will receive extra time for reading. UK28 is just slightly faster in reading, but rather poor in comprehension. However, since none of the values is below the threshold, the child will not be granted any extra time or assistance. With a mixed approach, these issues are better tackled, and all of the children that could benefit from a diagnosis may potentially get one.

**FIGURE 2:**
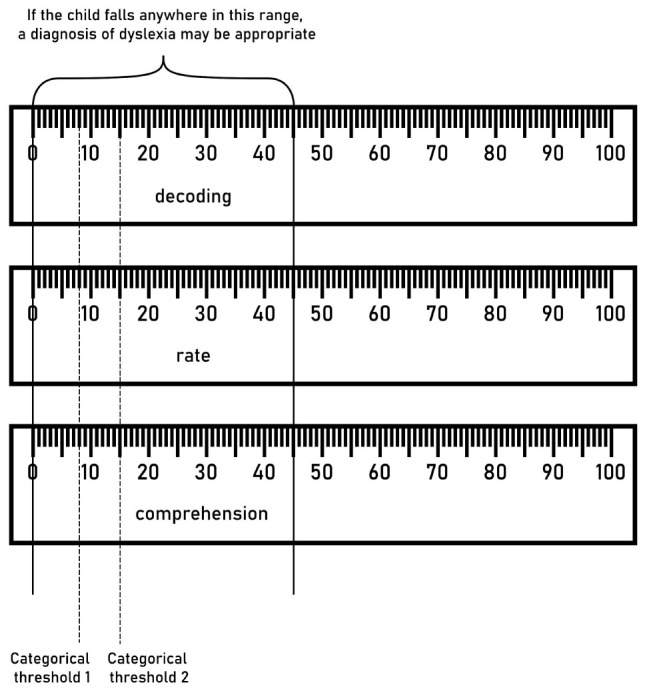
Multicomponent assessment chart. A chart of this kind could be included in reading assessments to help clinicians define a diagnosis.

One important aspect of the current proposal regards the combination of a graded assessment with a sub-categorical approach to dyslexia, as in Friedmann and Coltheart (2018). Such a combination practically consists of using the mixed approach described above and applying that to separate components of reading. If we consider the three main components of reading assessment to be decoding, speed and comprehension (Snowling et al., 2009), then each clinician should deal with a chart like the one in Figure 2. The chart can be interpreted as follows (if used on English):

A deficit in decoding might indicate phonological dyslexia (Lovett et al., 2000; Wolf and Bowers, 2000).

A deficit in speed might indicate surface dyslexia (Lovett et al., 2000; Wolf and Bowers, 2000).

A deficit in comprehension might indicate a specific reading comprehension deficit (Bailey et al., 2016).

Crucially, the notion of *deficit* would not be bound to the thresholds, but it would be dependent on the parameters described in Figure 1. According to this approach, both UK6 and UK28 may receive a diagnosis of surface dyslexia, since both show a deficit in reading speed. According to the current common practices (which favor a categorical approach), instead, they would not receive any.

## Discussion

The vast majority of assessments has a clinical aim, and for this reason, tests are designed to answer specific questions: does the child tested have dyslexia? Does the child qualify for assistance? Despite being pragmatically useful, these questions hide some problems. Plenty of evidence shows that reading performance in children shapes on a continuum (Shaywitz and Shaywitz, 2005), and it tends to the normal distribution (Snowling, 1998; Pennington, 1995)^2^. The diagnosis of dyslexia is based on a cut-off point on this continuum, and this cut-off point is arbitrary (Snowling, 2013). The reasons for using an arbitrary cut-off point are cultural and sociological, rather than scientific. It is necessary to get diagnoses (yes/no answers) in order for children to receive or not receive clinical assistance (Protopapas, 2019). Countries, which fairly differ in their regulations, need to develop policies that are available to children with a diagnosis and unavailable to children without a diagnosis (Stannard and Huxford, 2007).

Without denying the importance of *actually* having an assessment (Robin, 2006), the use of such a cut-off approach in the diagnosis of dyslexia poses some limitations. First, it does not lead to a faithful representation of the population (Pennington, 1995): A binary diagnosis of dyslexia may offer the illusion of a binary distribution of performance in children, with some children falling fairly low in the distribution (and thus obtaining a diagnosis), and the majority of children performing fine. However, a large number of children actually perform close to this arbitrary boundary, suggesting the presence of reading difficulties that are not “severe enough” to receive attention (Elliott and Gibbs, 2008; Barbiero et al., 2012; Protopapas, 2019). This is the case, for example, of UK5 and UK28 in the current study. Second, this approach makes accounts of the prevalence of dyslexia circular: 5% of children have dyslexia if one puts the cut-off point in the lowest 5% of the distribution, 7% have dyslexia if one uses 7% as a threshold, and so on. The notion of prevalence itself is thus an artifact of the assessment methods, and it does not reflect the population’s distribution.

An alternative possibility to categorical assessments would be to use graded assessments, where the diagnosis is not categorical (Elliott, 2006; Elliott and Gibbs, 2008; Protopapas, 2019). Elliott (2006), for example, proposes a system in which anyone who identifies as a poor reader, independently of where they fall in the distribution, would get into additional teaching streams. This approach, already observed in educational and clinical practice in some countries, offers two advantages: the first consists in assisting a larger group of children, since children in the gray area are more than those getting a diagnosis of dyslexia or those being flagged as being below average. Second, it avoids the social stigma of being diagnosed with a disorder, a stigma that seems even more illogic if one considers the actual distribution of reading skills in pupils.

The current paper showed that concerns for the limits of categorical assessments are legitimate, and some children have difficulties which do not get recognized with a categorical approach. This article offers a practical guideline on how to conciliate the classic categorical approach with a continuous approach. Following the method proposed here, children can get a “mixed” diagnosis, where some elements from categorical approaches are included, but a more graded gaze to each subcomponent of reading is adopted. As we showed in the analysis section of this article, with this mixed approach at least two additional children in our sample would receive attention (and possibly a diagnosis) in comparison to a classic categorical approach. In addition, the article shows that subtypes of dyslexia can easily be accounted for by applying this mixed method separately to the various subcomponents. A dissociated deficit in one component indicates the presence of a subtype of dyslexia.

In the current study, UK5 is a good example of the possible advantages of a mixed approach: With the classic threshold method he does not receive any attention and he does not qualify for special teaching streams, because he is not below average in any domain. With the system proposed in this article he would receive attention, since both rate and decoding are in the lower part of the distribution. His lowest score is in reading rate, and in a semi-opaque language such as English thus indicates the possible presence of surface dyslexia.

The rationale for this mixed approach is the following: Given a large enough sample, we expect a continuum of performance in each subcomponent of reading we decide to assess. Children falling in low or gray areas of each distribution, if struggling, should be considered for educational help, without flagging them as disordered. This approach could offer a practical pipeline to put in place proposals that lean toward a continuous assessment, such as Protopapas (2019). In the meantime, this approach can still identify differences such as those outlined by the distinction between phonological vs. surface dyslexia (Friedmann and Coltheart, 2018). Children in the low or gray part of the “speed/phonological” distribution would correspond to the classic diagnosis of surface dyslexia, while children in the low or gray part of the “decoding” distribution would correspond to the classic diagnosis of phonological dyslexia (if assessed in English).

In short, the present proposal boils down to the idea that a child may qualify for educational help if they meet the following criteria:

– They identify as poor readers
– They fall in low or gray parts of the distribution in at least one domain

Few final words may be spent on the cross-linguistic validity of these claims: The present article describes an English sample, and English is an interesting language because its orthography taps in both the phonological and the lexical routes (Coltheart et al., 2001). However, the approach to diagnosis proposed in this article has a “natural” cross-linguistic validity, because the components suggested for assessment are relevant across languages. In general terms, we expect different patterns across different languages. In transparent orthographies, speed/phonological processing are expected to have higher sensitivity, possibly showing larger variance, and based on previous work they are generally expected to be more effective in recognizing the presence of difficulties (Serrano and Defior, 2008). In very opaque orthographies we expect decoding to have a similar role (Chung and Ho, 2010). However, while it is possible that some domains will be particularly or more frequently vulnerable in some languages, the opposite patterns are not unattested, and it is important to have a system to detect them. For this reason, all domains should be included in the assessments across languages, if we aim at cross-linguistic validity, independently of the language used. It should be stressed that despite the potential cross-linguistic development of this method, further research is necessary, both on English and on other languages: The current study only involved British children, and in addition the sample presented is rather small and it comes from a geographically restricted area. Further assessments addressing a wider section of the English-speaking population, as well as assessments with similar design conducted on languages with different levels of opacity, are necessary, and they will help determine the validity of this approach.

## Data Availability Statement

The raw data supporting the conclusion of this article will be made available by the authors, without undue reservation.

## Ethics Statement

The studies involving human participants were reviewed and approved by the University of Cambridge Ethics Committee. Written informed consent to participate in this study was provided by the participants’ legal guardian/next of kin.

## Author Contributions

LC and IT conceived the study. LC collected the data, drafted the article, and completed the revision. IT contributed with advice and suggestions in all of the phases of the study.

## Conflict of Interest

The authors declare that the research was conducted in the absence of any commercial or financial relationships that could be construed as a potential conflict of interest.

## Footnotes

1 Website gov.uk https://www.gov.uk/government/statistics/income-and-tax-by-borough-and-district-or-unitary-authority-2010-to-2011#history

2 This is not surprising. Many variables, when plotted, shape as a normal distribution. In biology, for example, an approximate normal distribution is obtained when measuring height or weight of a large group of animals of the same species. In cognitive science, the measures of several domains of cognition tend to the normal distribution (for example, IQ, language skills or memory).
